# Case of Spontaneous Gangrene

**Published:** 1877-12

**Authors:** Abner Hard

**Affiliations:** Aurora, Ill.


### CASE OF SPONTANEOUS GANGRENE.

By Abner Hard, M. D., Aurora, Ill.

[Extracts from a Paper read before the Fox River Valley Med. Asso'n, July 2d, 1877.]

Cases of spontaneous gangrene are not of frequent occurrence in country practice, though occasionally met with as a result of embolism, or disease of some of the arteries or veins. The case to which I call your attention at this time, seems to possess unusual interest, from the many members involved and the apparent absence of arterial disease. In the medical works to which I have had access, I have been unable to find a corresponding case.

April 30th, 1877, I was summoned to attend Miss M. N., aged sixteen, small in stature, but of bright intellect; not physically developed more than girls usually are at the age of twelve or thirteen years. She has never menstruated, nor exhibited other signs of sexual maturity. Her father is living and healthy; her mother died at the age of thirty-seven, of uterine tumor. She has been living in the family of her aunt, and attending school for two years, having been always considered very healthy. During the past winter, she has been troubled with an urticarial eruption, appearing and disappearing, accompanied with itching. It did not make her ill, nor cause any uneasiness in the minds of her friends. On the 17th of April, she complained of headache, and did not attend school on that, nor on the following day. April 19th, the rash reappeared, and she took a cold water bath. The rash at once disappeared, and she felt sick and faint, but returned to school,

It reappeared each evening, until April 26th, when the ends of her fingers and toes became swollen, purple and painful. She came to Aurora and consulted a homœopathic practitioner, who called it "erratic erysipelas," and prescribed for her. She returned to school, but grew worse, and on Saturday, April 28th, Dr. Hawley, of Aurora, was called, and prescribed. She had no other medical attendance until my arrival, April 30th. I found her suffering excruciating pain in both hands and feet, which were black and swollen. The black color extended to the middle of the hands and feet, from which it shaded off into red, and then to the natural color. The extremities of the fingers and toes were senseless; but no line distinguished the dead from the living tissues. Except for a short distance from the ends of the extremities, the integument only was involved. A needle thrust into the tissues, beneath the skin, would give pain. She was very restless, and had not slept for several days. Pulse 120 per minute, and very distinct, with natural fullness. Heart-sounds not quite natural, but without irregularity. There was a peculiar double click to the first sound, while the second was natural. Tongue coated; bowels rather constipated; urine less abundant than usual, with a trace of sugar.

I prescribed one-fourth of a grain of morphine every three hours, to be given at short intervals if necessary. Quinia grs. iii; tr. ferri chlo., gtt. xv. every four hours; also fl. ex. digitalis, one minim every four hours. Feet and hands to be kept fomented with carbolic acid water, three grains of acid to the ounce of water. May 1st, I found my patient had rested pretty well, although when the morphine had been omitted, the pain returned. No other changes in the symptoms. Ordered a laxative and treatment continued. May 4th, bowels had moved properly, but the other symptoms were aggravated. Anodynes had not been continued. Pulse 130; heart-sounds about the same as at last visit, and the discoloration had extended. Prescribed tr. cinchonæ comp., and tr. gentian. in equal parts; a teaspoonful every four hours, in place of quinine. Fl. ext. digitalis, three drops every four hours. Continue iron and give morphine regularly to relieve pain. As the case progressed, I found as I increased the digitalis, the heart-sounds improved as well as the other symptoms, until the pulse decreased in frequency to (or below) 80 beats per minute, when its action became irregular, and the symptoms were worse. When the pulse was allowed to rise above 110, the patient was in a less favorable condition. She invariably improved when the pulse was kept between 80 and 100 per minute. The patient was brought to Aurora, May 21st, and upon May 25th, the line of separation being complete, in presence of Drs. Robbins, Groat, Higgins, Howell and Elliott, I amputated the toes and heads of all the metatarsal bones of the right, and all the toes, except the fifth of the left foot. May 28th, I amputated all the fingers, except the index finger of the right hand, and, June 19th, I completed the amputations by removing the thumb and index finger of the right hand, leaving, however, stumps of all the fingers (but one), which may become quite useful. The amputations were performed under the influence of ether, by completing with the knife the line of demarcation formed by nature; pressing back the granulating flesh, and cutting off the bone, except where the separation occurred at a joint, in which case I disarticulated. In no case was any living tissue removed, and the stumps were allowed to heal by granulation. The parts removed were mummified, and hard as bone. One of the fingers I here present for your examination.

The dressings for a few days after amputation, consisted of carbolic acid lotion applied daily, and an ointment composed of one drachm of boracic acid to the ounce of cerate, spread on strips of old cotton or linen cloth. Following the first amputations, the nose and ears became discolored, swollen, and painful, having the appearance presented by the fingers and toes, in the early stage of the disease, previous to my being called, and I feared they also would become gangrenous. But as the effects of the ether passed off, and the pulse beats numbered less than 100, the natural color, and condition of the parts were restored. The same condition of the nose and ears followed each succeeding amputation.

The case has progressed favorably under the persistent use of digitalis, and tonics, without the necessity of anodynes, to the present time, June 30th; the stumps being nearly all healed, and the patient gaining in appetite, flesh, strength, and spirits. Upon reviewing the case, I am led to the conclusion that the cause of the disease must be attributed to the central organ of circulation, the heart, as I could find no lesion to account for it; and the further fact, that when from any cause, either the omission or too free use of digitalis, or an increased action of the heart from the administration of ether, or the shock of amputation, the circulation was much disturbed, the pain, swelling, and tendency of the gangrene to spread, or new parts to become involved, was apparent. It will be observed that the parts affected were those most distant from the heart, and possessed of the weakest circulation: viz, the fingers, toes, ears and nose.

P. S.—Aug. 1, 1877. Since the above was reported, the patient has continued to improve, with no symptoms of a return of the disease. All the stumps have healed, and are well formed, and she walks, and feeds herself. The abnormal condition of the heart remains, though greatly improved. She still continues to take the preparations of digitalis, bark and gentian.

